# Supplementary material for: Drone-based medication delivery for rural, flood-prone coastal communities
Source: PLoS One. 2025 Oct 6;20(10):e0333696. doi: 10.1371/journal.pone.0333696 (PMC12500137; doi:10.1371/journal.pone.0333696)
Supplement: Attachment — Submitted filename: Response to Reviewer_medical delivery in ES June 30.docx [file pone.0333696.s002.docx]

**Response to Reviewer**

**Reviewer #1:**

1. This paper addresses the feasibility of using drone delivery for medicine compared to road transportation methods. The authors mostly emphasize on the reduced time as the most beneficial aspect.

Response: Thank you for your comment. We agree that reduced travel time is a key advantage of drone delivery over personal vehicle travel, and this was a primary focus of our GIS-based analysis. In the discussion section (*4.1*), we also expanded on additional benefits, particularly for elderly residents and their caregivers—highlighting the convenience of avoiding long, often burdensome trips from remote areas to pharmacies typically located in larger, more centralized communities along major roads.

2. During emergency situations, the reduced time is a crucial factor, but for general deliveries, maybe the role of reduced time is over emphasized. I think the study should have considered other crucial factors to make a solid case. Here are my observations:

Response: We have revised the manuscript accordingly. Please see our detailed responses below.

3. Though delivery time is reduced for a single delivery in drones, traditional vehicles can deliver in bulk and can complete multiple deliveries at neighboring region within a very short time thus significantly reducing overall time/delivery. Without accounting for this the comparison seems unfair. Other minor thing could be time to load from pharmacy to drone site shipment or similar issues.

Response: We agree that bulk deliveries via traditional vehicles can be an effective service model for rural communities. Bulk deliveries, however, may not serve all customers on the Eastern Shore. For example, residents of Tangier Island (included in the study area) are not connected to the mainland by any bridge or tunnel. Therefore, the only delivery methods currently available in that area are ferries and small airplanes. Our current analysis focuses on individual patients using personal vehicles to retrieve their prescriptions, rather than on pharmacy-led bulk delivery. As such, we did not include a direct comparison between bulk vehicle delivery and drone delivery.

To clarify this scope, we have revised the manuscript by specifying “personal vehicle travel” throughout the text. Additionally, we have added a new paragraph in the *Introduction* section highlighting the importance of personal vehicle use in rural areas, particularly for healthcare access. We believe these revisions help better define the study’s focus and provide readers with appropriate context for interpreting our findings. Please refer to: *“Personal vehicles are essential for mobility and access to critical services in rural communities, particularly for older adults. A national study of individuals aged 65–79 found that rural residents were 7% more likely than their urban and suburban peers to emphasize the importance of driving (Strogatz et al., 2019), underscoring their reliance on personal transportation. In rural North Carolina, individuals with a driver's license made 2.29 times more healthcare visits, while those with access to family or friends for transportation had 1.58 times more visits (Arcury et al., 2006), highlighting how transportation directly affects healthcare access. Yet, despite this heavy reliance, older drivers in rural areas face disproportionate barriers due to chronic health conditions, physical impairments, and age-related declines in driving ability (Lyman et al., 2001; Krasniuk & Crizzle, 2023). Without viable transportation alternatives, these challenges severely limit rural seniors' access to care and independence.”*

We also added several sentences in *Section 4.3*, *Study Limitations and Future Directions*, addressing the comparison between truck-based and drone delivery, as well as the consideration of loading times from suppliers:

“ *… or the inclusion of loading times from suppliers to drone stations*.”

“*Future research could also explore comparative analyses between bulk vehicle delivery and drone delivery in rural settings (e.g., Chiang et al., 2019; Kirschstein, 2020) to assess relative efficiency and cost-effectiveness. For example, Kirschstein (2020) examined the energy demands of diesel trucks, electric trucks, and drones, finding that while electric trucks generally consume the least energy, drones are particularly competitive in rural contexts. Such comparisons could support the development of more sustainable and equitable delivery strategies to address healthcare access disparities in rural communities.*”

4. There is no ballpark cost estimates/comparisons per delivery between the methods in the paper. While one driver can possibly deliver to many people in a day, a drone pilot might not be able to reach much people due to one shipment/flight requiring more skilled pilots. Other aspects like battery costs/replacement costs could challenge the financial feasibility as well. At least a rough estimate of costs/delivery would be helpful.

Response: We appreciate the Referee’s suggestion. As noted in a previous response, this study specifically focuses on comparing personal vehicle travel with drone delivery, rather than bulk delivery by truck. We have updated Introduction section, Page 6 to address the concern of delivery cost as follows:

1. As an estimate, a recent consulting report by PricewaterhouseCoopers estimated that the average cost of drone delivery per item was between $6 to $25. The cost is expected to drop to $2 per delivery by 2034 (Gajewska et al., 2024).
2. In the United States, Walmart offers drone delivery at certain locations for $19.99 per order (Walmart, 2024).
3. A drone delivery company called Manna that operates in Ireland has achieved a cost of $4 per order (Bogaisky, 2025).

It is important to note that a single drone pilot is currently permitted to operate three drones at the same time. The pilot observes the three flights remotely. In the future, the FAA may allow a single drone pilot to operate even more drones at the same time to help reduce the cost of deliveries.

5. This report is based on a particular location. How generalizable is this study to other places? Some discussions would be helpful.

Response: We have included a paragraph to address this comment. Please refer to section 4.2: “*The geospatial analysis framework developed in this study is designed to be scalable and generalizable to other regions. By relying exclusively on publicly available datasets from federal, state, and county sources (Table 2), the methodology protects patient privacy while ensuring broad applicability. Although parcel data was obtained locally to target residential land use, most other datasets, such as road networks, building footprints, and elevation, are widely accessible across the U.S. This allows for straightforward replication in other medically underserved or flood-prone communities, making the approach a flexible and resource-efficient tool for planning drone-based healthcare delivery in diverse rural settings*.”

6. I appreciate the authors for addressing a problem which might benefit several people get access to healthcare. I would appreciate more if case for drone based delivery is more convincing.

Thank you for your thoughtful comment. We agree that making a stronger case for drone-based delivery is essential to highlight its potential in addressing healthcare disparities in rural communities. In response, we have expanded the Introduction section to include additional context on the regulatory approval of drone-based medical delivery, as well as current cost estimates reported by major companies such as Walmart.

Furthermore, in *Section 4.3*, we have added a detailed paragraph discussing real-world test flights conducted in the study area by DroneUp. This includes over 350 actual and 90 simulated flights, demonstrating the operational feasibility and delivery speed of drones across various distance ranges. We also highlight feedback from patients who participated in the pilot program, which reflects strong community acceptance and the perceived usefulness of drone delivery services.

These additions aim to present a more convincing and evidence-based case for the viability of drone-enabled healthcare access.

**Reviewer #2:**

7. The abstract lacks quantitative evidence on how much drone delivery improves accessibility or reduces delays compared to traditional methods, which weakens the impact of its findings.

Response: Thank you for your comment, we have revised the description of abstract to include quantitative evidence. Please see the revised abstract: “*Compared to traditional vehicle travel, drone delivery reduced trip times from up to 50 minutes to under 10 minutes for more than 80% of the population, including elderly patients*.”

8. The proposed vulnerability indices are mentioned, but the abstract does not explain how they were validated or their effectiveness in prioritizing patients, leaving their practical utility unclear.

Response: We have added descriptions of the spatial analysis used to examine the clustering of vulnerable patients and the prioritized station. Please refer to the revised abstract “*These indices were examined using Getis-Ord Gi* spatial analysis, which identified statistically significant clusters of high-need patients, particularly around the northernmost drone station. The results reveal that elderly residents in remote, low-lying areas are especially vulnerable to missed prescriptions due to both transportation barriers and flooding.*”

9. The introduction should clearly conclude with a distinct paragraph that highlights the novel contributions of your work.

Response: We have revised the second last paragraph of introduction to highlight the novel contributions of our study. Please refer to the *Introduction*: “*Using Virginia’s Eastern Shore (hereafter ES) as a case study, this study makes three key contributions to the field of healthcare accessibility and disaster-resilient delivery systems. First, it quantifies the substantial time savings of drone-based medication delivery over traditional vehicle travel in rural, flood-prone areas, providing one of the first direct comparisons in a non-emergency context. Second, it introduces two spatial vulnerability indices that integrate demographic, geographic, and flood exposure data to identify high-need patients, offering a replicable framework for prioritizing healthcare interventions. Third, it evaluates drone hub performance and reach across varying vulnerability profiles, guiding more effective placement and resource allocation under operational constraints. Together, these contributions demonstrate the feasibility and strategic value of drone delivery networks for enhancing medication access in underserved coastal communities and offer a scalable model for broader implementation in similarly challenged regions worldwide*.”

10. The literature review should benefit from more explorations of previous studies.

Response: We have added a new paragraph highlighting the importance of personal vehicles in rural areas and the additional transportation challenges faced by older populations. We also incorporated the work of Tomio et al. (2010) to provide further evidence on how flooding disrupts access to medication, and cited a news article by Popper (2015) that documents the first government-approved medical drone delivery in Virginia. Please refer to the *Introduction* section.

11. The discussion section needs to be expanded to more thoroughly analyze the results.

Response: We have revised the discussion section to provide a more thorough analysis of the results, as requested. Please refer to *Section 4.1* for the updated content. In response to this comment, the word count for this section has been expanded from 552 to 831 words to offer deeper insights into the findings and their implications.

12. The first paragraph of the conclusion should succinctly summarize the contributions of the study in past tense.

Response: We have revised the conclusion to be more succinctly summarize the contribution in past tense. Please refer to the *Conclusion* section.

13. The second paragraph of the conclusion should provide clear and actionable future recommendations.

Response: We have included a new paragraph to provide clear and actionable future recommendations. Please see second paragraph in Conclusion section: “*Future research should enhance travel time estimates by incorporating real-world driving behaviors, dynamic traffic conditions, and more flexible drone routing systems. Improving the vulnerability scoring methodology by integrating health condition prevalence, behavioral factors, and community-specific thresholds could offer a more accurate assessment of patient needs. Addressing service gaps in no-fly zones through alternatives like truck-based drone delivery and conducting community surveys to guide strategic planning are also recommended. Finally, the geospatial dataset developed here provides a valuable foundation for future cost-benefit analyses and stakeholder collaboration to expand drone-based healthcare delivery in rural, flood-prone regions*.”

14. Some equations are not properly cited.

Response: We have revised the relevant sections to ensure that all equations are properly cited.

15. Please place the figures within the text for the next round of revision.

Response: We have inserted figures within the text.
