# Supplementary material for: Drone-based medication delivery for rural, flood-prone coastal communities
Source: PLoS One. 2025 Oct 6;20(10):e0333696. doi: 10.1371/journal.pone.0333696 (PMC12500137; doi:10.1371/journal.pone.0333696)
Supplement: Attachment — Submitted filename: Responses to reviewer Sept 4.docx [file pone.0333696.s003.docx]

**Response to Reviewers**

**Reviewer #2**:

1. Minor English proofreading is required, please look for typos as they do exist within the manuscript.

**Response**: Thank you for your comment. We have reviewed the manuscript and corrected identified typos; please refer to the tracked changes document for details.

**Reviewer #3:**

2. Title and Abstract
   • Title: Drone-Based Medication Delivery for Flood-Prone Coastal Communities
   • Abstract:
   Access to healthcare remains a critical challenge for rural populations, particularly in flood-prone coastal communities where transportation barriers limit access to essential medical services. This study evaluates the effectiveness of drone-based medication delivery in improving healthcare accessibility for vulnerable populations on Virginia’s Eastern Shore. Compared to traditional personal vehicle travel, drone delivery reduced trip times from up to 50 minutes to under 10 minutes for more than 80% of the population, including elderly patients. Using publicly available datasets, we developed two transportation vulnerability indices that incorporate age, travel time, and flood risk to prioritize patients for drone-based pharmaceutical delivery. These indices were examined using Getis-Ord Gi* spatial analysis, which identified statistically significant clusters of high-need patients, particularly around the northernmost drone station. The results reveal that elderly residents in remote, low-lying areas are especially vulnerable to missed prescriptions due to both transportation barriers and flooding. Our approach demonstrates how drone delivery can reduce healthcare access disparities while offering a scalable and resilient framework for other medically underserved regions, especially under time or resource constraints.
   Introduction
   • Aim and Motivation:
   o Aim and Motivation is stated in introduction part.
   o However, it should be explained little more.
   o More references and explanation should be added in introduction as the introduction is very less.

**Response**: We appreciate the reviewer’s comment. While the original introduction was intentionally concise to maintain focus, we have expanded it by adding 11 additional references and contextual details to strengthen the background and motivation for our study. Because the reviewer did not specify the missing literature, we incorporated studies across multiple dimensions to enrich the introduction, including:

- **Literature supporting research motivation**
  - *Casey et al. (2002)*: financial constraints affecting rural pharmacy services
  - *Berenbrok et al. (2022); Law et al. (2013); Todd et al. (2015); Sharareh et al. (2024)*: disparities in access to pharmaceutical services between urban and rural communities
  - *Tharumia Jagadeesan & Wirtz (2021)*: review of studies on pharmacy accessibility
  - *Ranković Plazinić & Jović (2018)*: mobility limitations of elderly populations
  - *Wassmer et al. (2025)*: impact of flooding on healthcare accessibility
- **Literature on medical delivery using drones**
  - *Snouffer (2022)*: pilot programs for drone-based medical deliveries
- **Literature highlighting existing gaps**
  - *Comi & Savchenko (2021); Garus et al. (2024)*: comparisons of delivery methods
  - *Berenbrok et al. (2022); Sharareh et al. (2024)*: methodologies for calculating driving times

We believe these additions provide a stronger foundation for the aim and motivation of our study, clarifying the significance of addressing rural healthcare access challenges and the potential role of drone-based solutions.

3. Research Questions and Objectives:
   o The research objectives are not mentioned in the paper, please mention the objectives of the paper.

**Response**: We have included the research objective in the last paragraph of the introduction. Please see “*The primary research objective is to develop and assess a scalable framework that integrates drone-based delivery, spatial vulnerability analysis, and operational modeling to improve equitable healthcare access in disaster-prone rural regions*.”

4. • Literature Review:
   o Some more related papers can be included in the related work section.

**Response**: Thank you for your comments. We have added 11 additional references to the introduction section; details are provided in our response to Comment #2.

5. Methodology
   • Clarity of Methods:
   o The methods are stated clearly that is good.
   • Innovativeness:
   o The proposed approach is novel.

**Response**: Thank you for your comments.

6. Results and Analysis
   • Presentation of Data:
   o Presentation of the data is good.
   o Some more details and references can be added in the introduction areas.
   o More details can be added in the other sections like methodology and conclusion sections.

**Response**: Thank you for your comments. We have added 11 additional references to the introduction section to provide more context and support; details are provided in our response to Comment #2. We have added more details regarding the methodology in the introduction section: “*this study compares traditional vehicle-based pharmaceutical delivery with drone-based systems in flood-prone coastal communities and evaluates patient vulnerability. Travel times were estimated for both delivery modes, and two vulnerability indices—one incorporating age and travel time (VAT) and another adding flood impacts (VATF)—were used to identify high-risk populations. Hotspot analysis guided prioritization of drone stations, demonstrating how drones can improve timely medication access for the most vulnerable residents*.”

We have also added more details about methodology into the conclusion:“… *this study assessed healthcare accessibility in flood-prone coastal communities by comparing traditional vehicle-based pharmaceutical trips with drone-based deliveries, while also evaluating patient vulnerability. Travel times for personal vehicles were calculated using a hierarchical road network and round-trip travel assumptions, whereas drone delivery times were estimated using direct flight paths from drone stations. Population layers were then aggregated within travel zones to quantify access efficiency. To identify the most at-risk populations, two vulnerability indices were developed: VAT, incorporating patient age and vehicle travel time, and VATF, additionally accounting for floodwater interruptions. Hotspot analysis identified statistically significant clusters of high vulnerability, guiding the prioritization of drone stations for service deployment. Overall, this integrated approach demonstrated how drones can improve timely access to medications, particularly for the most vulnerable populations, and provided a scalable framework for optimizing resource allocation under both everyday and flood-impacted conditions*.”

7. • Analysis and Discussion:
   o The results are well analyzed.

**Response**: Thank you for your comment.

8. Conclusion and Contributions
   • Summary of Findings:
   o The conclusion and future scope are stated good.
   o But state what innovative method can be used in the future.
   o State how your approach can help in the relevant field?

**Response**: In addition to a new paragraph on future directions in the Conclusion, we have edited the text to incorporate additional innovative methods: “*Future research should enhance travel time estimates by incorporating real-world driving behaviors, dynamic traffic conditions, and more flexible drone routing systems, including multi-drone networks, station exchanges, and variable drone speeds. Improving the vulnerability scoring methodology by integrating health condition prevalence, behavioral factors, and community-specific thresholds could provide a more accurate assessment of patient needs. Addressing service gaps in no-fly zones through alternatives like truck-based drone delivery, conducting community surveys, and assessing human-drone interactions are also recommended. Finally, the geospatial dataset developed here offers a valuable foundation for cost-benefit analyses and stakeholder collaboration to expand efficient, adaptive, and community-informed drone-based healthcare delivery in rural, flood-prone regions*.”

We have also added a statement regarding the contribution to the relevant field: “*This approach helps improve healthcare access by identifying high-need populations, optimizing drone hub placement, and providing a scalable framework for resilient, equitable medication delivery in underserved communities*.”

9. • Contributions:
   o The contributions to the field are stated nicely.
   o Some more details can be added in the conclusion part.

**Response**: We have included a paragraph regarding the main contributions of this manuscript in the conclusion section. Please refer to “*The three main contributions of this study include: first, quantifying the substantial time savings of drone-based medication delivery compared to traditional vehicle travel; second, developing two spatial vulnerability indices that integrate demographic, geographic, and flood exposure data to identify and prioritize high-need patients; and third, evaluating drone hub performance and geographic reach under varying vulnerability profiles to guide effective placement and resource allocation. Together, these contributions demonstrate that drone delivery networks are not only feasible but also strategically valuable as more equitable and resilient alternatives to personal vehicle–dependent healthcare access in underserved coastal communities*.”

10. Language and Writing Style
    • Grammar and Clarity:
    o Some very little mistakes in grammar, that can be revised.

**Response**: We have reviewed the manuscript and corrected identified typos; please refer to the tracked changes document for details.

11. References
    • Relevance and Recency:
    o The references are relevant.
    o However the references provided are very less, some more references can added. I have also recommended some references you can add them and extend the referencing area.

**Response**: Thanks for your suggestion. The manuscript included 83 references in the prior round of revisions; we added 11 more references to the introduction to further strengthen the context, as detailed in our response to Comment #2. Additionally, we incorporated one of the specific references the reviewer suggested, as noted in our response to Comment #14. With these updates, the manuscript now cites 95 references, which we believe provides thorough and sufficient coverage.

12. • Formatting:
    o References are in proper order.
    o However some more related references can be added, some references are given below in the recommendations section.

**Response**: Thank you for your comment. We have added 11 more references to the introduction to further strengthen the context, as detailed in our response to Comment #2. We have included a reference that the reviewer suggested. Please see our response to comment #14.

13. Figures, Tables, and Equations
    • Figures:
    o The figures should be explain in more details inside the text.
    • Tables:
    o The tables should be explain in more details inside the text.
    • Equations:
    o Explain each parameter of the equation/algorithm.

**Response**: Thank you for your feedback. Since the comment did not specify which details were lacking, we focused on Figures 5–8 and Table 5 in the Results section to ensure their explanations are more comprehensive and better integrated. We hope this addresses your concern and improves the clarity and readability of the manuscript. We also have included more detailed explanations for equations and their parameters. Please refer to the Result section and the descriptions for equation.

14. Recommendations for Improvement
    1. The introduction section can be furnished with some new papers like:.
    a. https://doi.org/10.4108/airo.5855
    b. Using the different AI methods : https://doi.org/10.1007/978-981-97-5979-8_8 , https://doi.org/10.1007/978-981-97-5979-8_7
    other food deliravy robot: doi: 10.1109/ACCESS.2024.3355278.

**Response:** Thank you for providing these insightful articles. We appreciate the reviewer’s suggestions and have carefully considered each recommended reference. The paper *“****Moshayed et al., 2024 Robots in Agriculture: Revolutionizing Farming Practices****”* has been added to section *4.3 Study Limitations and Future Directions* . This placement allows us to acknowledge its relevance to the broader field while keeping the introduction concise and closely aligned with the primary goals of our study.

We also reviewed the book chapters *“****Moshayedi et al., 2024 Meta-heuristic Algorithms as an Optimizer: Prospects and Challenges (Part I and II)****.”* However, we found it challenging to establish a direct connection between meta-heuristic algorithms and our current application, which focuses on spatial and vulnerability-driven modeling rather than algorithm optimization.

Finally, regarding *“****Moshayedi et al., 2024 Design and Development of FOODIEBOT****,”* we determined that food delivery robotics fall outside the scope of this manuscript, which is centered on healthcare-focused drone delivery in flood-prone rural communities. For this reason, we did not include this reference.

15. Please answer below question
    How did drone delivery compare to traditional personal vehicle travel times for medication delivery in Virginia’s Eastern Shore?

**Response:** Drone delivery was markedly faster. Over 80% of Eastern Shore patients could receive medications within 10 minutes by drone, whereas many remote communities faced vehicle round trips of 20-50 minutes to the nearest pharmacy. In places without practical vehicle access, such as Tangier Island, drones reduced delivery time to under 10 minutes and provided a viable alternative to boat or plane transport.

16. What factors were incorporated into the transportation vulnerability indices developed in this study?

**Response:** The vulnerability indices incorporated three main factors: patient age, vehicular travel time to the nearest pharmacy, and flood-related travel interruptions. Patient age was assessed by calculating the percentage of individuals aged 60 and older in each census block and assigning those values to building footprints. Vehicular travel time was determined by calculating the shortest driving routes from each building footprint to the nearest pharmacy. Flood-related interruptions were evaluated using FEMA 100-year flood zones and road networks to categorize each property’s access as not affected, detoured, blocked, or inundated. Two indices were created: VAT, which included age and travel time, and VATF, which added flood interruption to capture the additional impact of flooding on patient access.

17. Which area was identified as having the highest concentration of vulnerable patients, and why?

**Response:** The western coast of the Eastern Shore was identified as having the highest concentration of vulnerable patients. This area’s elevated vulnerability is largely due to its low-lying, flood-prone conditions, which, when factored into the VATF index, increased vulnerability scores and created concentrated hotspot patterns in the spatial analysis.

18. What role did community engagement and collaboration with multiple organizations play in the project’s success?

**Response:** Community engagement and collaboration were critical to the project’s success. Local stakeholders, healthcare providers, drone service experts, and academic researchers worked together to address complex transportation barriers and design an effective, data-driven drone delivery network. The Accomack-Northampton Planning District Commission facilitated community outreach, ensuring local needs were understood and addressed. Healthcare providers like Riverside Health guided the medical delivery requirements, while DroneUp provided the technical expertise and operations. Academic partners supported project management and data modeling. This transdisciplinary collaboration ensured that the system was both technically feasible and responsive to the community’s healthcare needs.

19. What future improvements to the vulnerability scoring methodology and service delivery were recommended by the study?

**Response:** We recommended refining the vulnerability scoring methodology by integrating additional factors, such as the prevalence of health conditions like hypertension and behavioral aspects related to driving patterns by age and gender, to more accurately capture patient vulnerability. We also suggested the need to account for chronic flooding risks, including high-tide flooding, which poses a more frequent and growing threat compared to the relatively rare 100-year flood events, especially with projected sea-level rise. Incorporating community-specific data, such as patient preferences and critical thresholds for acceptable travel times, through surveys or healthcare utilization records, was suggested to make the vulnerability assessments more locally relevant. Additionally, evaluating human-drone interactions to better understand community perspectives on security, usability, and acceptance could help refine and improve the effectiveness and equity of drone-based service delivery.

20. Overall Evaluation
    • Final Recommendation:
    • paper format and presentation should modify based on template.
    • Major Revisions.

**Response:** We have carefully revised the format for our manuscript according to the Submission Guidelines (https://journals.plos.org/plosone/s/submission-guidelines).
